# Chemopreventive Potential of Dietary Nanonutraceuticals for Prostate Cancer: An Extensive Review

**DOI:** 10.3389/fonc.2022.925379

**Published:** 2022-07-12

**Authors:** Hitesh Chopra, Shabana Bibi, Rajat Goyal, Rupesh K. Gautam, Rashmi Trivedi, Tarun Kumar Upadhyay, Mohd Hasan Mujahid, Mohammad Ajmal Shah, Muhammad Haris, Kartik Bhairu Khot, Gopika Gopan, Inderbir Singh, Jin Kyu Kim, Jobin Jose, Mohamed M. Abdel-Daim, Fahad A. Alhumaydhi, Talha Bin Emran, Bonglee Kim

**Affiliations:** ^1^ Chitkara College of Pharmacy, Chitkara University, Punjab, India; ^2^ Department of Biosciences, Shifa Tameer-e-milat University, Islamabad, Pakistan; ^3^ Yunnan Herbal Laboratory, College of Ecology and Environmental Sciences, Yunnan University, Kunming, China; ^4^ Maharishi Markandeshwar (MM) School of Pharmacy, Maharishi Markandeshwar University, Sadopur-Ambala, India; ^5^ Maharishi Markandeshwar (MM) College of Pharmacy, Maharishi Markandeshwar (Deemed to be University), Mullana-Ambala, India; ^6^ Department of Biotechnology, Parul Institute of Applied Sciences and Animal Cell Culture and Immunobiochemistry Lab, Centre of Research for Development, Parul University, Vadodara, India; ^7^ Department of Pharmacy, Hazara University, Mansehra, Pakistan; ^8^ Faculty of Pharmaceutical Sciences, Government College University, Faisalabad, Pakistan; ^9^ Department of Pharmaceutics, NITTE Deemed-to-be University, NGSM Institute of Pharmaceutical Sciences, Mangalore, India; ^10^ Department of Pathology, College of Korean Medicine, Kyung Hee University, Seoul, South Korea; ^11^ Department of Pharmaceutical Sciences, Pharmacy Program, Batterjee Medical College, Jeddah, Saudi Arabia; ^12^ Pharmacology Department, Faculty of Veterinary Medicine, Suez Canal University, Ismailia, Egypt; ^13^ Department of Medical Laboratories, College of Applied Medical Sciences, Qassim University, Buraydah, Saudi Arabia; ^14^ Department of Pharmacy, BGC Trust University Bangladesh, Chittagong, Bangladesh; ^15^ Department of Pharmacy, Faculty of Allied Health Sciences, Daffodil International University, Dhaka, Bangladesh

**Keywords:** cancer, chemotherapy, prostate cancer, nutraceutical, supplements, nanotechnology, nutrition

## Abstract

There are more than two hundred fifty different types of cancers, that are diagnosed around the world. Prostate cancer is one of the suspicious type of cancer spreading very fast around the world, it is reported that in 2018, 29430 patients died of prostate cancer in the United State of America (USA), and hence it is expected that one out of nine men diagnosed with this severe disease during their lives. Medical science has identified cancer at several stages and indicated genes mutations involved in the cancer cell progressions. Genetic implications have been studied extensively in cancer cell growth. So most efficacious drug for prostate cancer is highly required just like other severe diseases for men. So nutraceutical companies are playing major role to manage cancer disease by the recommendation of best natural products around the world, most of these natural products are isolated from plant and mushrooms because they contain several chemoprotective agents, which could reduce the chances of development of cancer and protect the cells for further progression. Some nutraceutical supplements might activate the cytotoxic chemotherapeutic effects by the mechanism of cell cycle arrest, cell differentiation procedures and changes in the redox states, but in other, it also elevate the levels of effectiveness of chemotherapeutic mechanism and in results, cancer cell becomes less reactive to chemotherapy. In this review, we have highlighted the prostate cancer and importance of nutraceuticals for the control and management of prostate cancer, and the significance of nutraceuticals to cancer patients during chemotherapy.

## 1 Introduction

Natural derivatives are excellent sources of bioactive composites and are widely distributed as the most efficacious modern medicines (1–3). In recent years, several researchers attained a lot of interest in the natural dietary agents, due to their therapeutic potential in cancer suppression and lowering the threat of cancer cell development (4). Nutraceuticals are characterized as an emerging food category that includes dietary components, and delivered the benefits to keep balance in health by improving nutritious standards. Nutraceuticals are predicted to have relatively lower toxicity and are associated with adverse effects as compared to traditional synthetic medications, which are used to cure identical symptoms since they are derived from natural nutritional resources; but have presented dose-dependent effects (5–11). Nutraceuticals act as an interface between, nutrition and pharmaceuticals (12). It may be challenging to consume the entire nutrients required for the maintenance of normal physiological and physical health. The amalgamation of novel nutraceutical derivatives with foodstuff are easy to consume and becomes functional foods for body (13).

**Figure 1:**
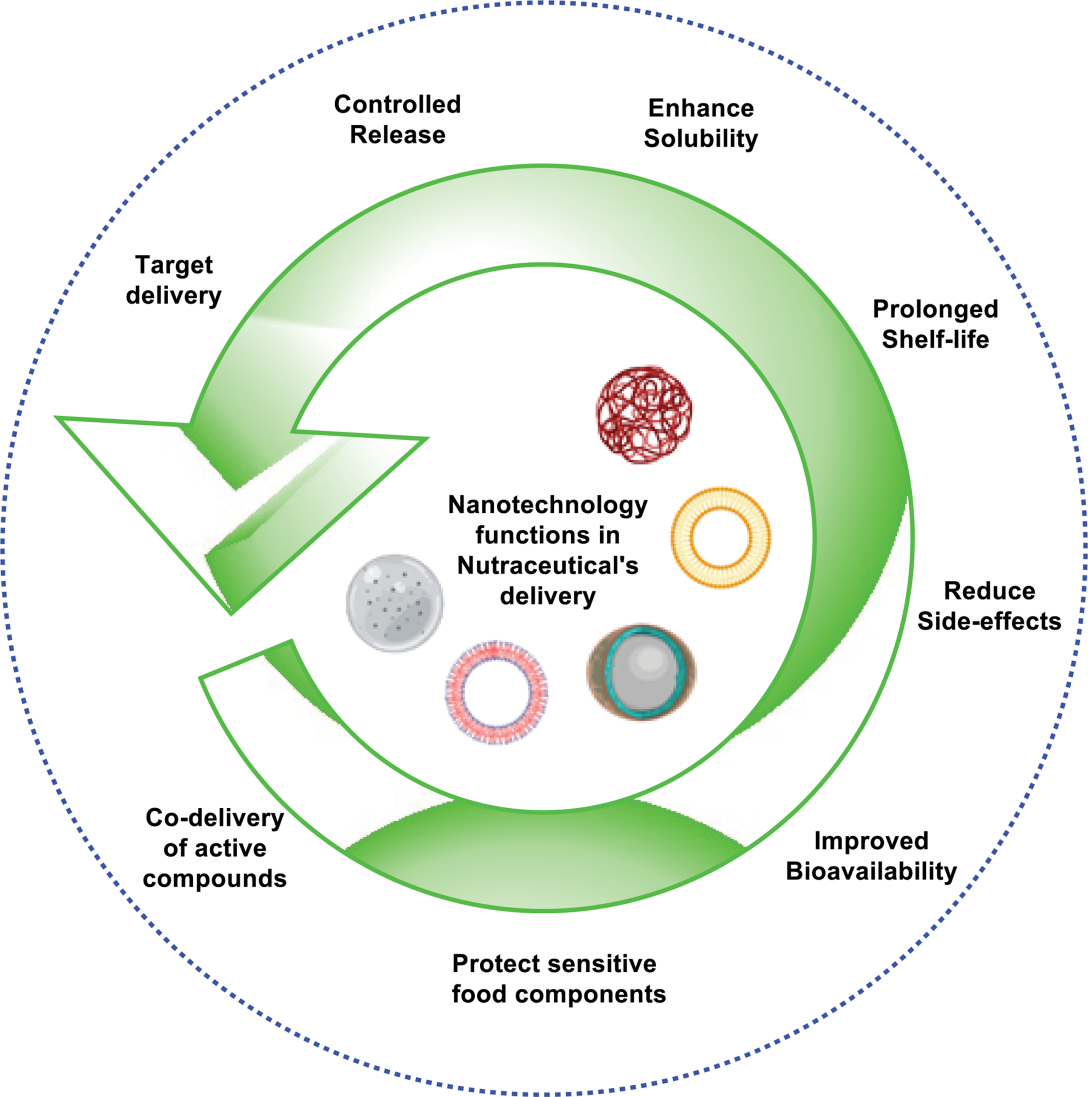
Role of nanotechnology in nutraceutical’s delivery system.

In recent decades, the combination between nutraceuticals and nanotechnology has received a lot of attention from several research organizations. Unfortunately, several nutraceutical products are of major concerned, because it has less benefits to health, due to their weak physicochemical characteristics such as poor absorption, less stability, lower water solubility, and probable chemical alterations after their administration. Nanotechnology could be considered as a breakthrough in activating the therapeutic characteristics of nutraceutical products for the human well-being as immunity booster and protect the body from forigen harmful entities. Variety of ailments based on their nutraceuticals potential efficacy and limiting bioavailibilty aspects. As a result, nanotechnology could be a new frontier in the development of novel supplementary nutritional products with less adverse effects and more health benefits (14). Several nutraceuticals such as quercetin, curcumin, coenzyme Q, thymoquinone, and green tea polyphenols have been delivered into nanoparticles and are effective in ‘‘nano chemotherapy” and ‘‘nano chemoprevention’’ (4). The various role of nanotechnology in the delivery of nutraceuticals is illustrated in **Figure 1**.

## 2 Cancer and Challenges Faced for Treatment Using Traditional Approaches

Cancer is a hazardous, life-threatening ailment having the utmost challenging afflictions worldwide (15). It portrays enormously alarming circumstances that are illustrated by unregulated cell growth, resulting in the incursion of nearby tissues and oftenly extending to other sections of body (16).

Regardless, the usual methodology of cancer treatment has been notclear; it relies on surgery, chemotherapy, and radiotherapy. Usually, radiotherapy and surgical resection are considered asoften successful techniques in the abolition of the primary tumor, even though disease relapse due to metastasis or residual cancerous cells is a ubiquitous problematic issue. As a result, chemotherapy is frequently used to address these issues (17).

There are a number of therapy options available to men diagnosed with localized prostate cancer—that is, illness that hasn’t progressed beyond the prostate region—based on the stage and grade of the disease (potential aggressiveness of the tumor). Some patients may have surgery on their own. Radiation treatment may be the sole option for some.

Some people may have both of these conditions at the same time. When there’s a fear that the operation didn’t remove all of the tumor tissue, this is a common reaction. Radiation treatment may also be advised if a patient’s PSA levels begin to increase months or even years after surgery, even if imaging has not been able to detect tumor development. A shorter and more intense course of radiation treatment after surgery is safe for many patients with prostate cancer. Aside from destroying or slowing the development of cancer cells, radiation may harm healthy cells in the area as well. Side effects are possible if healthy cells are damaged.

Radiation treatment often leaves its victims exhausted. To be fatigued, one must feel drained and worn out on a regular basis. When it happens, it might either come on quickly or gradually. If you’re having the same quantity of radiation treatment to the same place of your body as someone else, you may experience weariness in different ways.

The use of hypofractionated radiation therapy to treat prostate cancer has already been acknowledged by certain patients who are receiving radiation therapy alone for the disease. However, it’s not known whether this form of radiation treatment should be used after surgery.

There are several sensitive sites in your bladder and rectum that might be targeted by radiation following surgery. Radiation-damaged healthy cells often recover within a few months of therapy ending. It’s possible, though, that some individuals may have adverse effects that don’t go away. In other cases, symptoms may not appear for many months or even years after the completion of radiation treatment.

According to previous research it has been observed that several plant-based medicines play a vital role in molecular and cellular processes that underlie tumor development. Numerous chemotherapeutic agents for cancer treatment are produced from plants such as vinca alkaloids (e.g.,vincristine and vinblastine) and Taxol (*Taxus brevifolia*). Nutritional dietary agents could be advantageous in cancer treatment. Several evidences revealed that meals that are relatively lower in carbohydrates and higher in high-quality proteins, fats, and fibers are considered to be beneficial for cancer patients. In addition, nutraceutical products may also be advantageous in the reduction of toxicity and adverse effects allied with radiation therapy and chemotherapy, and provide improved living circumstances by plummeting tumor cachexia (18). This prevalent usage of nutraceuticals has receiveda lot of consideration for the importance of dietary nutrients in cancer pathogenesis (19).

## 3 Why Nutraceuticals Required for Cancer

Although numerous anticancer medications are available commercially, but the advent of acquired drug resistance, as well as the extreme side effects of these widely used treatments are main problems in efficacious chemotherapy. As a result, it is suggested that newer and innovative drugs have to be designed rationally with fewer adverse effects (20).

Nutritional dietary components and phytochemicals have a long and illustrious history, as well as substantial applications in the field of modern medicines. Nutraceuticals can influence DNA transcription and regulate the factors responsible for DNA damage in tumor cells (21). They have shown to re-sensitize drug-resistant tumors due to their pleiotropic property and capability to affect various signaling pathways (AMPK signalling pathway, EGF-mediated signalling pathways, NF-kB signalling pathway etc), which is a positive attribute of natural components. Nutraceuticals target the cancerous cells at multiple levels by acting on their molecular targets and cause cell cycle arrest or apoptosis by inhibiting the proliferation of cancer cells, and suppression of metastasis, invasion, or angiogenesis (22). They stop cancer from spreading by inhibiting the signaling pathways that are essential for cancer progression (23). For instance, Oleuropein reduces cell proliferation primarily through two mechanisms: on the one hand, it acts by inhibiting the cell cycle *via* upregulation of cyclin-dependent kinase (CDK) inhibitors, and on the other hand, it modulates the genic expression responsible for the induction of intrinsic and extrinsic pathways of apoptosis *via* the upregulation of p53 and p21. In addition, oleuropein may change the activity of critical molecules implicated in the initiation and progression of cancer, including MAPKs, the c-Met proto-oncogene, and the fatty acid synthase (FASN) enzyme (24). Demidenko et al. found that luteolin inhibited cancer cell proliferation throughout the G1/S and G2/M stages by inhibiting the HT-29 cell cycle (25). In addition, luteolin inhibits the overexpression of certain antiapoptotic proteins in afflicted cells and regulates the expression and activity of CDC2 (CDK1) kinase and cyclin B1 proteins, which trigger the G2/M transition phases in luteolin-treated colon cancer cell lines.

Many nutraceutical products such as soy isoflavones, curcumin, resveratrol, indole-3-carbinol, lycopene, green tea polyphenols, epigallocatechin-3-gallate, and 3,3’-diindolylmethane (DIM) cause downregulation of signal transductions such as Akt, PI3K, NFkB, mTOR and other pathways that are required for cancer progression (26). Some of them has been tabulated as **Table 1**.

**Table 1:** Tabulated data of studies showing nutraceuticals having anti prostate cancer activity.

Nutraceuticals	References
Curcumin	(27–32)
Genistein	(33–41)
Ellagic acid	(42–49)
Berberine	(50–57)
Piperine	(58–62)
Fisetin	(63–70)
Pomegranate	(71–73)
Delphinidin	(74–76)
Daidzein	(77–84)
Gambogic Acid	(85–88)
Lycopene	(89–98)
Luteolin	(99–108)
Isothiocyanate	(109–115)

**Figure 2:**
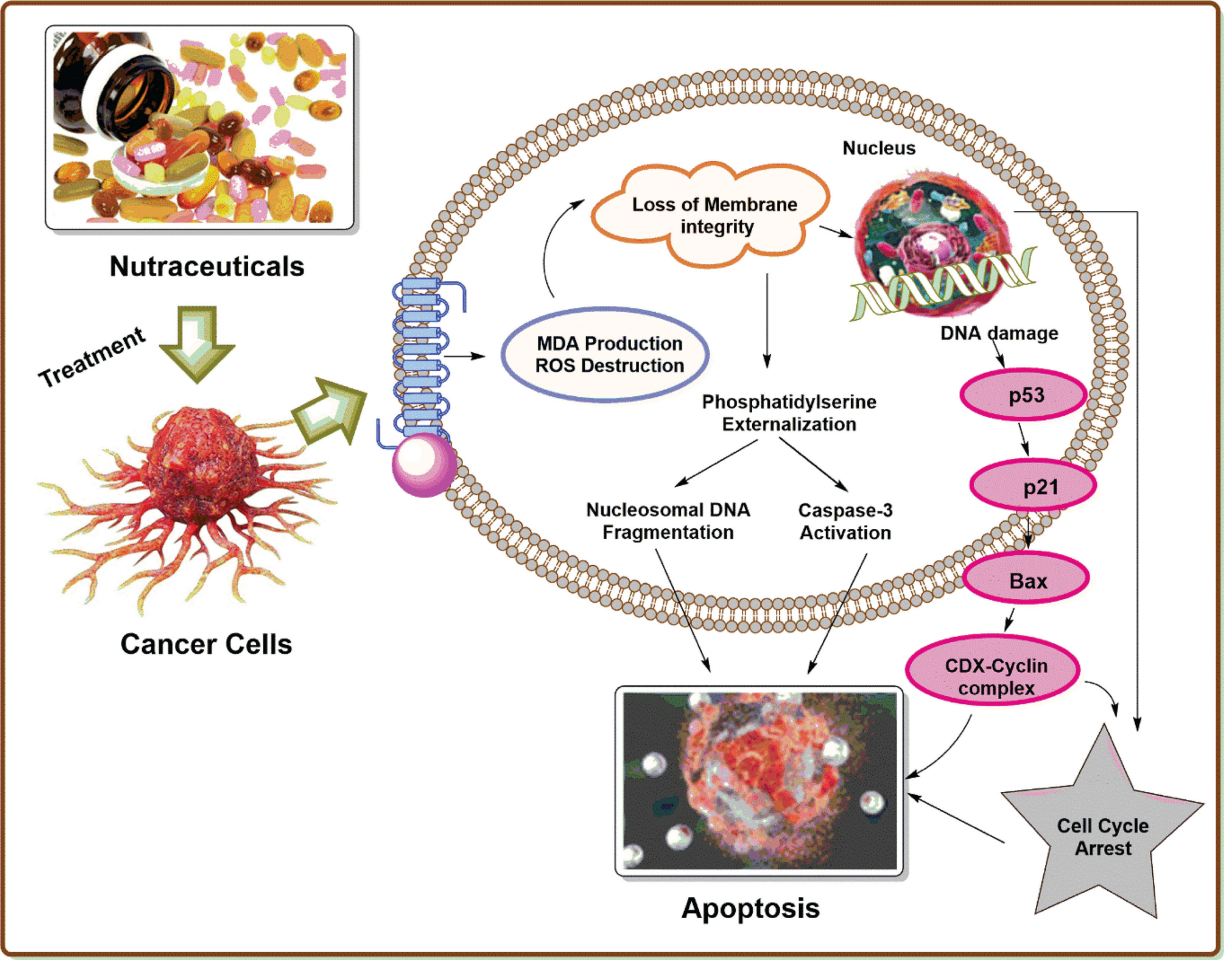
Molecular targets and mechanism of action of nutraceutical products in prostate cancer therapy.

Nutraceuticals have a great potential to modulate various molecular targets, such asgrowth factors [e.g., epidermal growth factor receptor (EGFR), insulin-like growth factor-1 receptor (IGF-1R), HER2, and VEGFR], transcription factors [e.g., STAT3, NF-κB, NRF-2, activator protein (AP-1), HIF-1α and peroxisome proliferator-activated receptor (PPARγ)], protein kinases [e.g., Bcr-abl, phosphoinositide 3-kinase (PI3K), Raf/Ras and AMPK], inflammatory mediators [e.g., TNF-α, 5-LOX, COX-2, CRP, IL-6, IL-8, and iNOS], and other targets that are involved in cancer progression (116). These therapeutic characteristics make nutraceuticals good candidates for suppressing carcinogenesis and improving treatment results in cancer patients (22). The molecular targets and mechanism of action of nutraceutical products in prostate cancer therapy are depicted in **Figure 2**.

## 4 Prostate Cancer

Cancer is the leading cause of death worldwide, where the mortalilty rate of cancer is increasing dramatically. Majority of the cancer cases in the world is represented by prostate cancer (PC) with an estimated value of 13.5% (117). PC is well known as a non-skin cancer and is considered as the most common form of male cancer in the world (118). The abnormal growth of cells from the prostate gland leads to the development of PC. It is slowly growing cancer that spreads tumor cells to the other parts of the body, especially to the bones and upper lymph nodes. The major risk factor for the development PC in men is their age, race, and family history of disease. In the first stage, PC is less pronounced. In the later stages Lower Urinary Tract Symptoms (LUTS) including pain and urinary incontinence, presence of blood in the urine, back pain, and pain in the pelvis region have been reported (119). The risk of death from the PC is less malignant as the slow growth of tumor cells is not fatal to the patient, and hence patient can survive with proper and effective treatment strategy. Deaths from PC occur in the metastasis stage, which is the worst part of cancer, where it spread to almost every organ of the body, including the spine, rectum, brain, bone, and lymph nodes (120). In general, PC is rare for the people below 50 years of age. The average age of the patient with PC is between 72-74 years, and about 85% of patients are diagnosed with it above the age of 65. Due to the genetic predisposition, the incidence rate of PC is high in families when compared with other forms of cancer. About 10-15% of patients diagnosed with PC will have at least one relative with the disease, and the first relatives of patients with PC are two to three times more likely to be affected with it. According to GLOBCAN 2020, PC is another common cancer after lung cancer affects men worldwide, including an estimate of 1,414,259 young people and 375,304 deaths by 2020. The incidence and mortalilty rate of PC in the world is associated with increase in the age on diagnosis (121). The incidence rate of PC increases with age, where among 350 men one under the age of 50 are diagnosed with PC. In every 52 males 1 between the age of 50-59 increases the incidence of PC, even 60% men above the age limit of 65 also increases the incidence rate of PC (122). The mortality rate of PC increases with the age and approximately 55% of deaths occur above the age of 65.

PC is mainly associated with the prostate gland. The prostate gland is a part of male reproductive system that produces alkaline prostatic fluid to maintains the health and function of the sperm (123). The prostate grows and matures quickly under normal circumstances as circulating androgen levels rises during adolescence (124). The prostate can be prone to inflammation, hyperplasia, and cancer, that can alter testosterone-regulated growth and function (125). The entire structure of prostate has been altered by cancerous growth due to the effects of increasing levels of androgens (126).

PC is caused by environmental factors, diet, hormones, lifestyle factors, and a person’s genetic history (127). PC is mainly related to the western lifestyle, especially foods high in fat, meat, and dairy products. PC occurs due to the abnormal growth in cells of prostate gland. This tumor growth is followed by initial mutations and genetic mutations including the p53 gene and retinoblastoma, ultimately leading to tumor progression and metastasis. As PC invades the area, abscesses in the temporal area spread to the neck of the bladder, and the peripheral-zone abscesses extend into the ducts and seminal vesicles. About 90% of PCs are adenocarcinomas. Squamous cell carcinomas make up less than 1% of all prostate carcinomas. In PC, 70% come from the surrounding region, 15-20% from the central region, and 10-15% from the temporal area. Most importantly, PC has multifocal and coordinated involvement of many prostate sites due to clonal and nonclonal cancer cells.

The treatment of PC and its recommendation rely on most of the factors which includes, possible side effect, type, stage of cancer, patient’s preferences, and overall health condition. Along with all treatments, patients should be monitored closely to demonstrate clinical, biomedical, and radiological progress. Repeated photography and baseline scanning throughout 3–6 months is highly recommended as a major challenge in determining appropriate treatment (128). The treatment policy of PC covers three approaches- radiation, surgery, and chemotherapy. The pharmacological agents available for the treatment of PC include antineoplastics, systemic antifungals, chemotherapy modulating agents, endocrine monoclonal antibodies, corticosteroids, bisphosphonate derivatives and radiopharmaceuticals (129). Docetaxel chemotherapy was the first treatment that showed improvement in PC. Survival benefits have been seen in all age groups of patients affected with PC, followed by the established form of docetaxel injected three times a week in 10 cycles as first-line chemotherapy. The side effects of docetaxel are similar to those seen with other medications such as nausea and vomiting. The main drawback of this drug is that it is associated with motor and sensory peripheral neuropathy. Moreover, testing for men with recurrent disease is higher, especially after more than six months of relief from docetaxel exposure (130). Cabaxitaxel is another PC chemotherapy medication, approved by FDA. The main drawback of this drug in patients receiving toxic substances such as neutropenic sepsis cannot tolerate the side effects produced by this drug. Concomitant steroids and antiemetics are offered to reduce the side effects produced by Cabaxitaxel (131). Abiraterone which falls under hormone therapy, is a selective inhibitor of cytochrome p450 17A1 (CYP17). The drug Abiraterone is available in the form of oral dosage form and is given in combination with prednisolone in low doses. The common side effects of this drug include an increase in mineral corticoid levels, leading to hypertension, hypokalaemia, and fluid retention (132). A major risk factor for this drug is high levels of transaminase, which may interfere with liver function over time. Enzalutamide is a novel antiandrogen that has shown significant antitumor activity before and after PC chemotherapy. The limitation of enzalutamide is the occurrence of seizures reported in less than 1% of patient treated with it (133). Radium-223 is a radiopharmaceutical compound known as alpharadin to treat PC (134). The drug attracts double-stranded DNA, breaks down nearby cancer cells simultaneously, and saves normal tissue without significant visual effects. One of this drug’s most common side effects is bone pain because it is not recommended for patients with arthritis. Sipuleucel-T is the only immunomodulatory agent approved to treat PC and the first FDA-approved medical vaccine. The main limitation of this drug is that it affects sexual and reproductive problems in patients (135).

### 4.1 Pathophysiology for PCa

Androgen receptor (AR) signalling is crucial for prostate differentiation and function, as well as PC development and progression. A single copy gene on the X-chromosome encodes the human AR protein (Xq11.2-q12). It is a 919-amino-acid protein that may vary in length due to poly-glutamine, poly-glycine, and poly-proline repeats of varying lengths. The length of poly-glutamine repeats has been linked to receptor activity levels. The length of the repetitions varies from 9 to 36 residues, with an average of 18 to 22 repeats. Spinal and bulbar muscle atrophy are linked to very lengthy repetitions (136, 137). Although there is some indication that the length of the poly-glutamine repeat is linked to the risk of PC, epidemiological studies have revealed no substantial link (138).

AR, like other nuclear receptor family ligand-activated transcription factors, has three main domains: an amino-terminal transcriptional activation domain (NTD), a DNA-binding domain (DBD) with two zinc finger motifs that determine the DNA sequences recognised by the receptors, and a carboxyl terminal ligand-binding domain (LBD) that provides the regulatory switch by which androgens control the receptor’s transcriptional activity. A nuclear localization signal is seen in the hinge region (H), which joins the DBD with the LBD. High-affinity DNA binding is also facilitated by a portion of the hinge region (138).

**Figure 3:**
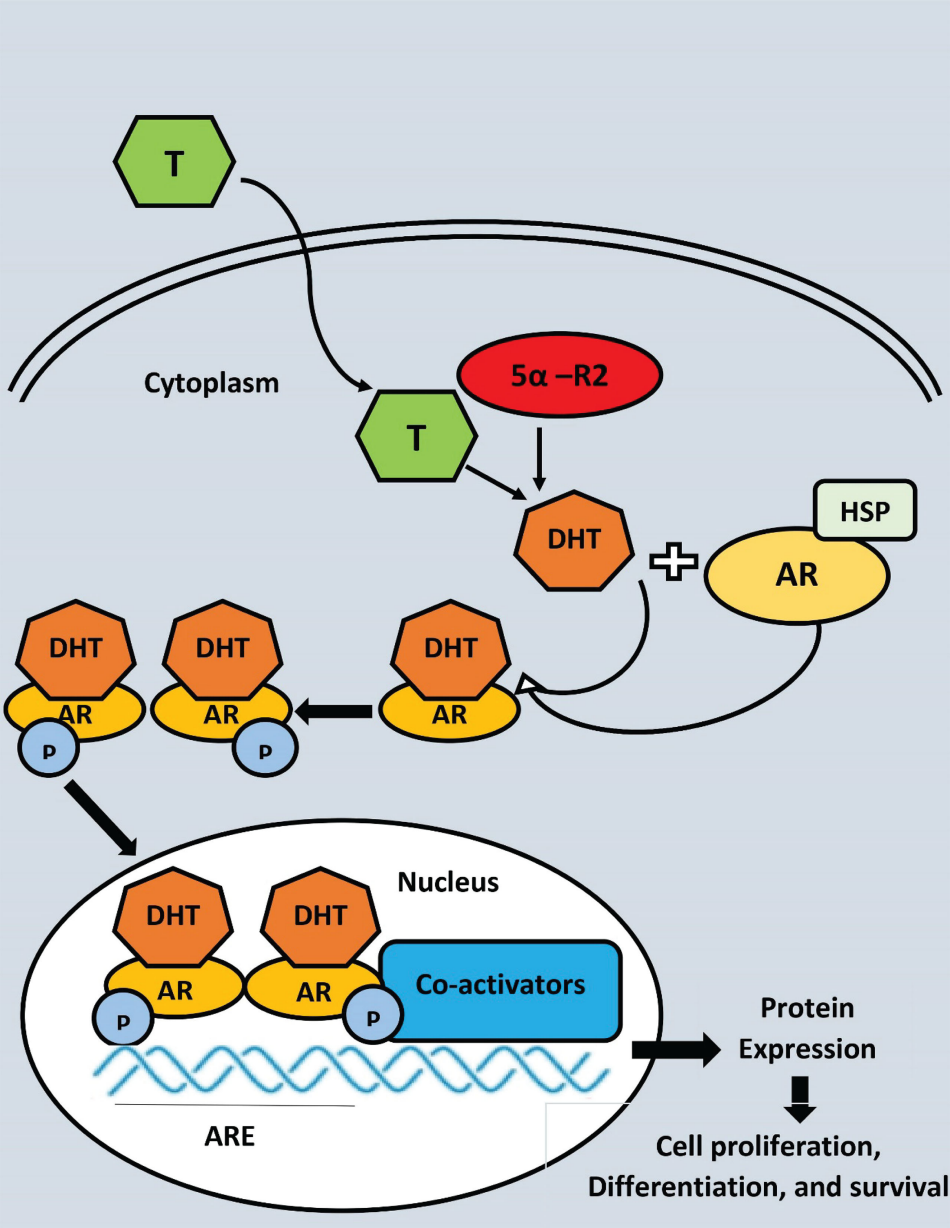
Androgen receptor (AR) ligand-dependent gene transactivation mechanism 5-reductase converts testosterone (T) to dihydrostosterone (DHT) in theprostate epithelical cell. DHT binding to AR causes dissociation of the AR-HSP (Heat shock protein) complex, dimerization, and nuclear translocation. AR binds to androgen response elements (ARE) in DNA and recruits co-activators to enhance transcription, where P, Phosphate group.

Androgen-receptor (AR) protein is stabilised and protected from degradation by heat shock proteins when androgens aren’t present (**Figure 3**). The two primary ligands of the AR, testosterone and dihydrotestosterone, control its activity (DHT). Prostate 5-reductase converts testosterone into the more powerful metabolite DHT, which is generated by testicular Leydig cells. In terms of AR binding affinity, DHT has a nearly 10-fold advantage over testosterone. The phosphorylation of numerous serine residues occurs as a consequence of DHT binding to the AR. Protection against proteolytic degradation, stability, and transcriptional activation are all possible outcomes of AR phosphorylation (139). AR transactivation is regulated by a number of coregulatory proteins that are able to react to changes in the microenvironment to control particular gene targets that are critical for cell growth and survival (140). There is a natural balance between cell proliferation and cell death in the normal prostate epithelium, but this equilibrium is broken in PC, resulting in tumor development (141).

### 4.2 Nanonutraceuticals Based Approaches for Prostate Cancer

Nanotechnology deals with wide range of technologies, materials, and production processes for the development of many medical products. The origins of nanotechnology create a variety of opportunities in various fields, maximum benefits are especially observed in the field of nanomedicine. Over the past two decades, the successive generation of nanoscale science and nanotechnology has been responsible for significant growth in the field of nanomedicine. At present, nanotechnology offers various benefits in the development of novel anticancer drugs that help to increase the immune system strength as compare to traditional medicine. Drug treatment associated with PC depends on the severity of the disease, especially the two types of methods have been considered for PC treatment. In the first case, early detection and pharmacotherapy are recommended and other forms of pharmacotherapy after surgical removal or radiation treatment has been recommended (142). Conventional treatment with the use of drug cells and nanotherapy may not work as the body tissue of the tumor patients and drug resistance varies greatly while simultaneously increasing the dose leads to systemic drug accumulation and toxicity. Therefore, it is an urgent requirement of improved drug treatment by increasing specificity by reducing systemic toxicity. The development of controlled nanocarriers with improved safety and efficacy, which could meet the clinical requirements for disease intensification and the production of a suitable clinical protocol has been widely accepted. With the discovery of newly identified approaches to relevant clinical challenges, nanotechnology plays a vital role in the treatment of PC (143).

The benefits listed above can be listed as targeted drug delivery for tumors, the onset of apoptosis, and drug accumulation in targeted tissues to increase the exposure of cancer cells (144). Despite the complex nature of the need for a natural origin for drug possibilities, studies began to focus on the possible additional use of dietary products that could be used to prevent, treat (or) delay the onset of certain health problems (145). Nutraceuticals, a compound name derived from ‘nutrients’ and’ medicines’, are defined as’ Phyto complex if their origin comes from the diet of vegetables and secondary metabolites when found in animal foods, are concentrated and mistreated (146). The most effective and promising way to support nutraceuticals’ health benefits is by the development of nutraceuticals at the nano level. Nanonutraceuticals provide greater safety and efficacy when used to treat health conditions, especially in those patients who cannot afford standard medical treatments. In addition to conventional therapy, a diet high in vegetables and fruits was associated with PC, suggesting that the disease could be prevented to some degree by changing lifestyle (or) eating habits (147). Lycopene, the primary carotenoid in tomato, has been linked to a reduced risk of prostate cancer, and preclinical studies have showed encouraging findings in normal prostate tissue showing tomato and lycopene may suppress androgen signaling. Live-cell Raman microscopy was used by Scarpitti et al. to study the transport of lycopene into PC-3 prostate cancer cells (148). In order to overcome lycopene’s low aqueous solubility and the difficulty of replicating physiological uptake by cells, the tween 80 micelle mimics natural lipoprotein complexes that deliver lycopene *in vivo*. It also provides a stable signal for assessing cellular uptake of the nutraceutical formulation. The Raman pictures show the lycopene’s subcellular distribution in the cells. At 532 nm, the Raman signal for lycopene is resonantly amplified, enabling a simple, sensitive, and label-free method to detect and quantify lycopene absorption in live cells. A reduction in local androgen regulatory signals and the production of insulin growth factor type-1 (IGF-1) and interleukin 6 were shown to enhance the rate of necrosis in mice prostate cells when lycopene was applied (149). For example, antioxidative, cell progression, apoptotic and insulin growth factor type 1 inhibitors of lycopene have been identified (150–152). In the past, researchers have shown a link between rising IGF-I blood levels and an increased risk of cancer, particularly prostate cancer. In transgenic rats, a higher frequency of prostatic intraepithelial neoplasia (PIN) was associated with higher levels of IGF-1 expression on the prostatic epithelium. As a result, IGF-1 was not only linked to an increased risk of prostate cancer, but it was also playing a role in carcinogenesis by promoting cell proliferation and interfering with the process of cell death (153, 154). Lycopene’s ability to suppress IGF-1 might be a major factor in preventing prostate cancer.In the field of targeted medication delivery, liposomes are the most widely used and thoroughly studied nanocarriers. Stabilizing therapeutic chemicals, overcoming barriers to cellular and tissue absorption, and enhancing biodistribution of compounds to target areas *in vivo* have all contributed to better therapeutics for a variety of biomedical applications (155–158). There are liposomes that have distinct aqueous regions that are made up of one or more concentric bilayers of lipid. Liposomal vesicles are able to encapsulate a wide spectrum of medications because of their unique capacity to encapsulate both lipophilic and hydrophilic molecules. There are hydrophilic and hydrophobic molecules in the aqueous core of the bilayer membrane A variety of macromolecules, including as DNA, proteins, and imaging agents, may be delivered through the vast aqueous core and biocompatible lipid shell (159–169). Drug delivery systems like liposomes have a broad variety of biophysical and physicochemical features that may be manipulated to alter their biological qualities. Particle size, charge, the number of lamellae, the lipid content, and surface modification with polymers and ligands all influence the stability of liposomal formulations in both *in vitro* and *in vivo* (170–177). Due to their natural phospholipid composition, liposomes are commonly thought to be pharmacologically non-toxic with minimal side effects. However, a growing number of studies have shown that liposomes may not be as immune-inert as once thought (178–183).

The use of liposomes in medicine opens up a world of therapeutic possibilities for a broad variety of diseases. Research into lipid-based medication delivery has grown significantly in the experimental *in vitro* and *in vivo* phases in the last 50 years since liposomes were first discovered. The use of liposomes in the administration of a broad variety of therapeutic and diagnostic substances and agents, such as drug molecules, gene therapy, and bioactive agents, is well-established in the field of liposome technology. There are several ways to increase the effectiveness of these formulations, including as altering the lipid composition, the charge of the lipid, and the inclusion of surface coatings or ligands (184–194).

*Curcuma longa* (curcumin) is a well-studied nutraceutical derived from the turmeric plant in its purest crystalline form (195) and has been carefully described in several studies. Various signalling pathways, including mitogen-activated protein kinase (MAPK), epidermal growth factor receptor (EGFR), and nuclear factor B (NFB), have been found to suppress PC cell proliferation and invasion and cause apoptosis *in vitro* and *in vivo* (196–198). Genes involved in inflammation, cell proliferation, and cell survival are heavily regulated by the NF-kB transcription factor. Many NF-kB-regulated genes, including as COX-2 (Cyclooxygenase-2), 5-LOX (5-lipoxygenase), TNF (Tumor necrosis factor), IL-6 (Interleukin 6), and EGFR tyrosine kinase activity, have been shown to be inhibited by curcumin (199–202). As part of the steroid receptor family, androgens play a critical role in the development and progression of PC (203, 204). To promote PC aggressive growth, aberrant activation of androgen signalling is caused by AR mutation and amplification (205). ARs and AR-associated cofactors have been shown to be suppressed by curcumin (206, 207). NKX3.1 (NK3 Homeobox 1), KLK3/(PSA) (Kallikrein related peptidase 3/Prostate-specific antigen), TMPRSS2 (Transmembrane serine protease 2), and TMPRSS2 (Transmembrane serine protease 2) were all downregulated by curcumin in both androgen dependent LNCaP and androgen-independent C4-2B cells. Nearly 90% of cancer-related fatalities are the result of metastasis (206, 207), which is a condition characterised by rapid cell proliferation. Understanding that several cell signalling pathways are disrupted in PC growth and bone metastases, the majority of PC medicines target particular targets. Thangapazham et al., formulated a liposomal formulation of curcumin to enhance curcumin’s anticancer activity against PC (208). The liposome of curcumin composed of dimyristoyl phosphatidyl choline (DMPC) and cholesterol as a primary ingredient for its preparation. The average particle size of liposomes was found to be 100-150 nm. When cells were exposed to DMPC liposomal curcumin (5-10 M) for 24-48 hours at 37 C, cell growth was 70-80% inhibited. Free curcumin, on the other hand, only showed comparable inhibition at levels ten times greater (>50 M). LNCaP cells were likewise shown to be more responsive to liposomal curcumin-mediated inhibition of cell growth than C4-2B cells. Curcumin liposomes and free curcumin both have a positive effect on LNCaP and C4-2B cells, with 31 and 70 percent of LNCaP cells surviving 10 M liposomal and free curcumin therapy, respectively. PC cell growth was inhibited more effectively by DPPC and DMPC liposomal curcumin than free curcumin. However, DMPC liposomal curcumin was shown to be the most effective of the liposomes examined.

Phan et al., prepared genistein loaded liposomes and stealth liposomes (GenLip) as a novel nanocarrier to enhance the solubility, bioavailability, pharmacokinetic properties, and cytotoxicity of genistein for specific induction of apoptosis in breast, ovarian and PC (209). The conventional and stealth liposomes containing phospholipid and cholesterol boosted genistein’s solubility, stability, and extended-release profile. The antioxidant activity showed peroxide neutralization in fluorescent probe oxidation assay quantitatively and microscopically for GenLip. The anticancer activity of GenLip was performed in a murine and human cancer cell line in a concentration and time-dependent manner. They performed the pro-apoptopic activity whereGenLip has maximum P53-independent apoptotic pathway markers compared with all treatments (209).

Silibinin and cabazitaxel based liposomes were prepared by Mahira et al, using ethanol injection based approaches (210). Lipids along with silibinin and cabazitaxel were dissolved in ethanol solvent, and TPGS was added with constant stirring to form liposomes during evaporation of solvents. The liposomes thus prepared has particle size of 100nm and showed enhanced antitumor activity on PC cell lines, indicating the potential for co-loading the molecules. The Hyaluronic acid based liposomes interfered in the G2/M cell cycle arrest causing apoptosis. The presence of HA caused increased in delivery of entrapped molecules into the CD44 expressing cells, and suppressing them (210).

The EMT, STST3, and AKT pathways, all of which are necessary for the evolution of PC, were inhibited by plumbagin therapy in the PTEN deletion PC mouse model, as described by Hafeez et al. (211). Oncogenic cells need high glucose absorption in order to fulfil their energy and anabolic demands in order to maintain fast proliferation and angiogenesis; as a result, cancer cells overexpress the GLUT transporter family, which consists of 14 members (212). Glucose transport in cells is increasingly being implicated in oncogenesis and tumor suppression, according to mounting evidence (213). This suggests that GLUT receptor expression might be suppressed in order to better our knowledge of the illness as well as to decrease tumor development. Genistein, a naturally occurring isoflavone, has been shown to have several health advantages, including anticancer properties (214). Genistein has been shown to increase apoptosis in hepatocellular carcinoma (HCC) *via* inactivating GLUT1 and thereby reducing aerobic glycolysis. PC cell lines were investigated by Chandler et al. for the presence of GLUT1 and GLUT12 mRNA and protein (215). The GLUT1 and GLUT12 proteins were found in the plasma membrane and cytoplasm by immunofluorescence. Tumors in the prostate, both benign and malignant, have various GLUT proteins (216). Experimentation with genistein on PC cells has demonstrated that Bax expression is increased, apoptotic signals are stimulated, and the anticancer effect of Cabazitaxel is enhanced to inhibit castration-resistant PC growth, as previously reported (mCRPC). The combination of genistein and cabazitaxel in the treatment of mCRPC xenograft tumors was shown to have a substantial effect on the growth of the tumors (217). Genistein’s action on cancer cells is restricted to the blockage of GLUT receptors, preventing glucose absorption, according to previous studies. As a result, administering it alone may not be sufficient to stop the spread of PC cells. It is thus possible to use genistein in conjunction with well-known anticancer medicines to better target cancer cells and achieve a higher therapeutic index. Tian et al., have developed liposomal preparation containing genistein and plumbagin for targeting delivery to PC specific membrane antigen (218). The Genistein Plumbagin liposomes (GPL) with size 80-100nm size, were conjugated with PSMA specific antibodies. The Plumbagin released rapidly from liposomes in comparison to Genistein. Plumbagin showed sensitizer effect on genistein, thus improving the anticancer activity and inhibiting the pristatae cancer. The GPL showed better effect on the LNCaP cell lines in comparison to PC-3 cells, which can be due to high expression of PSMA on LNCaP cell lines. The GPL increased the presence of free radicals and decreased expression of GLUT-1 receptors and Akt3 proteins. These events led to inhibition of proliferation of PC cells.

Unprocessed olive fruit and leaves are high in the natural component oleuropein (OL), which is a significant member of the secoiridoid family. Chemically, it is an elenolic acid/dihydroxyphenylethanolheterosidic ester that hydrolyzes to eleonolic acid and hydroxytyrosol (219), among other beneficial compounds. A variety of pharmacological actions have been found to include cardioprotection (antiarrhythmic), hypotensive (spasmolytic), and anti-inflammatory characteristics in OL. In addition, a human pharmacokinetic investigation found that oral administration of olive phenolic compounds resulted in fast absorption, metabolism, and renal clearance. Oleuropein’s bioavailability and metabolism were also shown to be very variable and depending on formulation factors as well as gender (220). As a result, intravenous injection is often recommended to address the drawbacks of oral delivery. However, no research has been done in animals to illustrate the pharmacokinetic characteristics of OL following intravenous administration. The drug is likely to have little impact on prostate tissue owing to its quick metabolism and elimination. A long-circulating intravenous approach is needed to circumvent the problems encountered by the oral route and to properly target PC cells with an effective dose of OL in PC care. By using a conventional film-hydration approach, the liposomal formulation was produced and extruded to produce nanosized vesicles with a limited range of sizes (221). In the passive targeting of cancer cells, increased permeability and retention (EPR) effect plays a significant role. The fenestrations in cancer cell membranes are 200 nm greater than in normal cell membranes, which typically have fenestrations of 50 nm. It is conceivable that nanocarriers that are between the lengths of 50 and 200 nm will find their way into cancerous tissues. At addition, the weak lymphatic system in the tumor site results in extended nanocarrier retention (222). It has been shown that cancer cells have a negatively charged surface owing to the release of lactic acid by cells with low oxygen levels (223). This liposome exhibits a positive zeta potential because cholesterol was incorporated into the lipid bilayer, which contains a positive charge head group. It was hoped that the liposomes’ positive charge would aid in tumor retention. This low positive potential may, however, lead to less stability due to decreased repulsion between charged particles. A substantial inter-bilayer repulsion was given by the presence of PEG on the liposome surface, which would have prevented aggregation (224). Comparative DSC thermograms of OL and OL-FML revealed a reduced peak of OL in the liposomes, which indicated that OL molecules were mostly contained inside the vesicular core. Liposomes’ polar surface and the presence of hydrophilic PEG strands make it possible for certain OL molecules to be adsorbed on their surface. Within the first hour of the investigation, 27.54 ± 2.995 percent of OL was released from the OL-FML, a burst release effect was detected. After then, there was a steady discharge over the next 24 hours. An aqueous soluble drug’s release pattern was seen in the OL solution. During the first hour of the experiment, the majority of the OL was excreted. The anticancer effectiveness of compounds and drug delivery systems is first determined *via* basic studies on cell viability. Researchers found that OL-FML has significantly inhibited cell viability in comparison to OL at all of the concentrations tested. The IC50 of OL-FML was much lower than that of OL solution. Cellular surface adsorption of liposomes and subsequent endocytosis are facilitated by the attraction of positively charged liposomes to negatively charged cell surfaces. Apoptosis and growth-promoting signals may be activated in cancer cells by persistent oxidative stress (225). In addition, OL inhibits Akt signalling by downregulating pAkt (226), which in turn leads to the activation of apoptosis in cancer cells. In the SH-SY5Y cell line, OL was tested for *in situ* TUNEL of nicked DNA and shown to induce apoptosis (227). TUNEL test has demonstrated that both OL and OL-FML induce apoptosis in 22Rv1 cells.

Zhou et al, developed curcumin-metal ion based liposomal formulation (228). The flower shaped confirmation of liposome was reported first time and effect of various metal ions were evaluated on the cancer cell lines. Metal and ligand selection was critical to the success of the cancer treatment medication complexes (229). Endogenous metal ions, which included a variety of trace metals, were not harmful to normal cells and were engaged in several metabolic activities. This study looked at the effects of various metal ions on the activities of drug metal complexes using the cations copper (Cu^2+^) and zinc (Zn^2+^). Because of verstality of curcumin, it was selected as a ligand in cancer treatment. Despite the Curcumin potential as an antitumor drug, its low bioavailability makes it less effective (230). Curcumin chemical stability in extreme physical conditions was greatly enhanced by complexation with metal ions (231). In spite of this, the Curcumin metal ions complex’s limited water solubility was a hurdle to its implementation. It was initially hoped that liposomes pre-loaded with metal ions solutions would be used to generate the Curcumin metal ion complexes. Intravenous injections might be used to administer the complexes produced in the liposomes. Curcumin metal ions complex liposomes’ characteristics will be affected by the kind of salt solution used to dissolve the metal ions (Cur-M liposomes). The liposomes seemed to change colour after the liposomes have become Cur-M. For example, liposomes containing copper or zinc exhibited a greater electrical conductivity (EE) than those that did not. Using Ca(Ac)2 liposomes, the Curcumin precipitated and retained its original colour when the trapping agents were Ca(Ac)_2_. Lipid liposomal formulation increased EE by increasing Chol content, while decreasing size. According to earlier studies, Chol content have presented opposite impact on hydrophobic medicines (232, 233). It was hypothesised that Chol’s interaction with hydrophobic medicines would reduce hydrophobic medications’ retention. As long as liposomes retain their rigid structure, the reactions between Cur and metal ions are made easier by adding high ratio Chol to them. It was noted that the EE of liposomes reduced when the drug to lipid ratio was more than 1:5. PBS (pH 7.4) with or without 10 mM EDTA was used for Cur-M liposomes release profiles. Cur-M liposomes, on the other hand, it has a more gradual release than Cur solution. There was a noticeable difference between Cur-Cu liposomes and Cur-Zn liposomes in terms of their structure. EDTA has no effect on liposomal release of Cur from Cur-Zn complexes. Cu-Cu liposomes’ Cur release may be substantially increased by mixing with EDTA. Cur-Cu complexes were shown to be more durable than Cur-Zn complexes to remain in solution over time. While Zn^2+^ ions remained in the liposome, Cur dissociated from Cur-Zn complexes. Liposomes containing Cur-Cu complexes progressively released the compounds. It was difficult to estimate the amount of released insoluble Cur-Cu complexes. Because of the trans-chelation reaction of the EDTA, the Cur was released from the Cur-Cu complexes. More than two times as long as the Cur-Zn liposomes, the t1/2 of Cur-Cu liposomes was 11.67 ± 4.45 h. With the Cur-M liposomes and FBS, Cur’s retention was also evidently different. Less Cur-Cu liposomal leakage in the early phase (0–12 h) was associated with a smaller change in Cur-Cu liposomal size. It was used as a trans-chelator in media and biological contexts. Because of its increased stability, Cur-Cu liposomes may function better than Cur-Zn liposomes in the bodily circulation when targeting tumors. Cur-M liposomal carriers combine the advantages of both coordination and encapsulation, and as predicted, they preserve Cur against degradation more effectively than earlier techniques. Due to the presence of serum proteins, liposomal formulations generated by the passive loading method during blood circulation are not able to protect Cur against degradation during blood circulation. As different formulations were taken up, so did the ROS level. This study found that after 2 h, the ROS level was greater in Cur solutions, and at 8 h it was higher in Cur–M liposomes treated groups. ROS production was mildly induced by both Zn^2+^ and Cu^2+^ liposomes. Cur-Cu liposomes were a little more effective in generating ROS than Cur-Zn liposomes. Metal ions have been found to interact with GSH, consuming GSH and influencing the ROS/GSH equilibrium, resulting in ROS production (234). In cancer cells with high GSH expression, the release of Cur from Cur-M liposomes would be accelerated, resulting in an increase in ROS production. GSH and other thiols, such as Cur, have been shown to bind covalently to ROS (235). Products of GSH-Cur conjugates that cannot be reversed, and may lead to oxidative stress. Cur’s cytotoxicity against cancer cells was enhanced by the cooperation of Cur and metal ions, which generated ROS. Cur-M liposomes were compared to Cur solutions in a subcutaneous tumor model and a lung metastasis model. For intravenous administration, 20 mg/kg of Cur was dissolved in ethanol and Tween 80. The Cur-Cu liposomes, on the other hand, outperformed other groups in terms of tumor inhibition. Cur-Cu liposomes may have a long-term anticancer impact because of the increased concentration of Cur in the tumor and the prolonged release of Cur to achieve this effect. The liposomes’ toxification-reactive complexes were potent in their ability to inhibit tumor development. Cur-Zn liposomes outperformed Cur solutions in terms of anticancer efficacy and circulating stability, as well as tumor tissue accumulation. Even though the Doxil-treated group have presented higher therapeutic outcomes, the lowest safety of Doxil revealed the downsides of chemotherapy. Though Cu^2+^ liposomes showed no toxicity to 4T1 cells when tested, they were shown to have a mild inhibitory impact on tumor development, equivalent to that of Cur solutions. As a result of this discrepancy, it was hypothesised that Cu may make a metalloenzyme inactive in metalloproteins, which were thought to be crucial to cancer cell metabolism through Zn replacement (229, 234, 236). In the meanwhile, Cur-Cu liposomes may have a greater impact on cancer treatment than Cur-Zn liposomes because of this.

**Figure 4:**
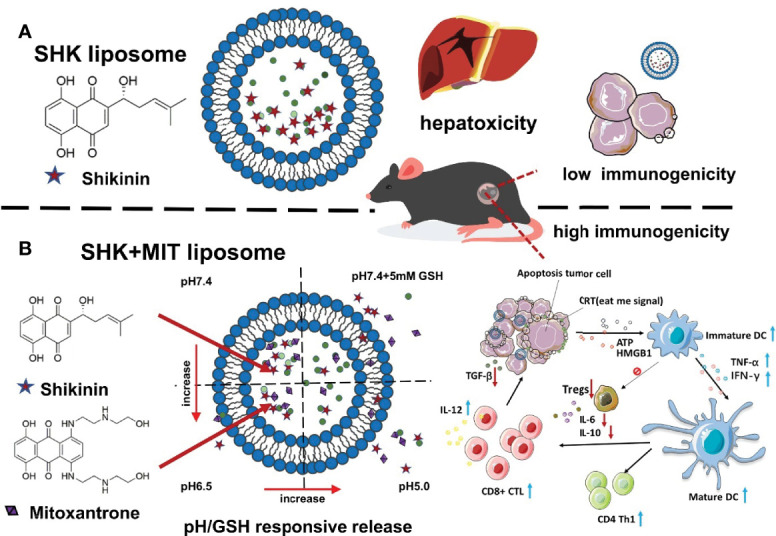
SHK and MIT co-loaded liposomes with dual-responsive release for synergistic chemo-immunotherapy through ICD. **(A)** Result of limited immunogenicity, SHK liposomes may cause hepatotoxicity. **(B)** Inducing strong apoptosis in cancer cells and stimulating an ICD effect and adaptive immune response using SHK and MIT co-loaded liposomes might shrink tumor volume and produce strong apoptosis in cancer cells.

Shikonin (SHK)was encapsulated as liposome moiety, to induce immunogenic cell death, at high dose (237). But loading resulted in hepatotoxic effect, so inorder to circumvent this issue, anthracycline mitoxantrone and doxorubicin were co-loaded to liposomes, for inducing the synegestic effect on tumor cells. A metal ion gradient was selected as the inner phase to stabilise SHK because it possesses the functional group necessary to form complexes with divalent metal ions (as shown in **Figure 4**). Cu^2+^ and Zn^+^ were found to be the only metal ions that could be successfully encapsulated in the first step of the experiment. Liposomes containing SHK-Zn were shown to be unstable because, after a few hours, a purple sediment of SHK-Zn developed. High-transition-temperature (HTT)-satiated phospholipid HSPC was chosen to increase the stiffness and stability of the lipid bilayer (237). Chol increases liposome size and decreases loading efficiency because hydrophobic drugs interact with Chol and get caught in the lipid membrane readily. According to earlier studies, reducing Chol concentration improves the retention of hydrophobic drugs (232). SHK was leaked when 10% Chol was being used. In order to avoid the other formulations’ instability, neutral Cu-gluconate was chosen to operate as the inner phase because SHK was successfully protonated and interacted with copper more closely in the neutral state than it did in the acidic condition (232). It was also shown that the greater the copper ion concentration, the more medication was loaded into the liposome and the more stable it was. To avoid copper toxicity, a threshold value of 200 mM was established for future study. Low levels of DSPE-PEG2000 (0.5 percent, molar ratio) were utilised to avoid ABC (accelerated blood clearance) and increase stability (238). It is necessary to utilise an organic solvent in order to enable SHK penetrate the bilayer; DMSO was employed at 5% and the drug/lipid ratio was 0.125. Its structural isomer alkannin was also employed to assess its encapsulation into liposomes throughout the formulation’s optimization process, yielding an unexpected result given that alkannin could not be loaded into liposomes despite the tiny structural difference.

#### 4.2.2 Nanoparticles

The increased permeability and retention (EPR) effect allows nanoparticles to deliver medications to tumors more effectively (239). A polymer core and a lipid shell form the core of lipid-polymer hybrid nanoparticles (LPNs) (240). Hydrophilic and hydrophobic pharmaceuticals may be encapsulated by the polymer core, which is covered by a lipid shell that acts as a barrier to prevent fast drug leakage and extend the release time (241). A recent study suggests that LPNs are an important component of combined PC treatment (242). Aptamer-functionalized LPNs have received the most attention from researchers in the development of ligands-functionalized LPNs for the treatment of cancer (243, 244). CUR and cabazitaxel (CTX) were conjugated ligands that were used to construct a curcumin and aptamer-functionalized hybrid lipid polymer nanoparticle (APT-CUR/CTX-LPN) (29). Each of the APT-CUR/CTX-LPNs measured on average at 121.3 nm in diameter and have noticed an electrically positive surface charge of 23.5%. PEG-PLGA or APT-PEG-PLGA nanoparticles, did not slow down drug release as much as LPNs, perhaps because of the presence of lipids. For sustained circulation, PEG is by far the most essential moiety because of its low immunogenicity and toxicity, as well as its great flexibility and little impact on the biological characteristics of medications that have been decorated (245). Because the pharmaceuticals released from APT-CUR/CTX-LPNs were slower than those released from CUR/CTX-LPNs, the aptamer on the surface may serve as a molecular barrier to keep the medications inside NPs (246). The cytotoxicity of LPNs was investigated in PSMA-positive LNCaP cells and PSMA-negative PC3 cells, both of which expressed PSMA. This may be due to the low cytotoxicity of blank APT-LPNs, which were composed mostly of biocompatible aptamer, SPC, PEG, and PLGA components (247). As a result, the cytotoxicity of the systems is caused by the drugs loaded into the LPNs. Evidence that LPN delivery methods may improve drug toxicity was found by comparing the cytotoxicity of medicines put into LPNs with that of drug solutions (248). PSMA positive LNCaP cells were more sensitive to APT-CUR/CTX-LPNs than PSMA negative PC3 cells, which suggests that APT-CUR/CTX-LPNs have the capacity to target PSMA positive cancer cells (249). In this work, the Chou-Talalay approach was used to evaluate the synergistic effects of the combination medication delivery system. CICUR+CTX values were lowest when the CUR/CTX ratio was 2:5, supporting the synergistic effect and pointing to the ratio in the APT-CUR/CTX-LPNs formulation. CUR has been shown to interact with a number of proteins that are involved in angiogenesis, metastasis, and cell survival, as well as disrupting dysregulated signaling pathways in cancer cells, such as PI3K/Akt and NF-kB. To increase CTX effectiveness in PC-3 cells, CUR increases the activity of many key enzymes including COX-2, NF-B, phospho-Akt, PI3K, and RTK (250, 251). More APT-CUR/CTX LPN dispersion was seen in tumor tissue than in CUR/CTX LPN or medication solutions. Gu et al. observed that aptamer-conjugated nanoparticles accumulated more in the tumor than unconjugated nanoparticles. Modifications to aptamers may explain this phenomena by delivering medicines to PC cells and causing tumor inhibition effects (252). CUR/CTX-LPNs have a significant antitumor activity compared to medicines. That the lipid outer layer has a strong affinity for the cell membrane and has merged with the cells and allowed medications to enter the cells (253) might be evidence of this. Experiments using APT-LPNs and 0.9 percent water demonstrated anorexia and anxiety in the mice, which might be the cause of their weight loss. Drug-loaded LPNs showed no noticeable changes in body weight, ALT, LDH, or BUN, indicating that severe adverse effects of anticancer treatment, have been adequately relieved while considerable co-therapeutic benefits had been preserved (254). Thipe *et al.*, designed resveratrol-conjugated gold nanoparticles (Res-AuNPs) as an innovative green nanotechnological approach to enhance the efficiency of bioactive phytochemical substance in cancer treatment (255). The study was planned to utilize the pro-apoptotic properties of gold nanoparticles (AuNPs) with synergistic antitumor properties of resveratrol as a green nanotechnological approach in cancer therapy. Res-AuNPs was coated with gum Arabic for stability of AuNPs. The particle size of Res-AuNPs and Res-GA-AuNPs was found to be 16.1 ± 5.0 and 14.9 ± 4.4 nm with negative zeta potential of -25 and -22 mV. The *in vitro* anticancer effect of resveratrol conjugated gold nanoparticle was conducted on human breast, pancreatic, and PC cell lines (MDAMB-231, PANC-1, PC-3). The results of their study on cancer cell lines provide evidence, which signify that increased corona of resveratrol on AuNPs improved the bioavailability of therapeutic active moiety in cancer cells.

Co-delivery systems for curcumin (CUR) and bioenhancement (B), “trikatu,” have been developed by Sharma et al. to treat hormone resistant PC (255). Spices such as black pepper (*Piper nigrum* Linn.) and long pepper (Piper longum Lind.) containing the active ingredient piperine (*Zingiber officinale* Rosc.) are known as “trikatu” in Japanese. Natural bio-enhancers combined with established anticancer medications in a delivery system may lead to higher bioefficacy and bioavailability, as well as expanded surface area, rescue of bioenhancers from degradation, and specificity in the treatment of tumors. Peeking into the drug transport pathway in PC3 cells using the neutral red test reveals an 18.4 percent increase in CUR influx from the inside of the cells. Bioenhancers like piperine, which inhibits the PGP, and gingerol, which affects cell membrane permeability and changes membrane dynamics are notable for this property (256, 257). To validate drug distribution, FITC-loaded nanoformulations were stained with DAPI for nucleus staining, and the remarkable impact was attributable to CH as a nanocarrier with pluronic F68, however CH conjugation with FA made the effect much more evident. Curcumin’s cellular internalisation is inhibited when given in conjunction with piperine in a nano-delivery method, providing further evidence that polyphenolic components like piperine and gingerol-6 modulate the transport mechanism. Additionally, it increases the CUR oral bioavailability (257, 258). Folate-conjugated nano-delivery systems may be increased since folic acid receptors are expressed on intestinal epithelial cells. FA enters cells by caveolae-mediated endocytosis and decreased folate carrier separate routes *via* facilitated transport, although the two pathways are not mutually exclusive (259). The synergistic impact of the polyphenolic bioenhancer in the formulation significantly reduced the IC50 of the CUR-B-CH-NPs in a cytotoxicity investigation using the PC cell line PC3. Unlike other cancer cell lines, the AR-negative PC3 cell line possesses very aggressive cells (259). To further understand the role of CUR-B-CH-NPs in hormone-independent PC, researchers extended studies on PC3 cells. Although CUR has been shown to be effective against AR arbitrated malignancies, it has some issues with cell absorption and bioavailability even in formulations designed for oral delivery (260). Apoptosis studies on PC3 cells showed that CUR-B-CH-NPs increased the percentage of apoptotic cells by 2.3 times higher as compared to CUR alone. It was discovered that the polyphenols in CUR-B-CH-NPs have an important role in apoptosis, since a complex mix of bioenhancers may activate distinct apoptosis-related pathways. The JC-1 dye based assay revealed a change in mitochondrial membrane potential, which is a key component of apoptosis. MMP levels were lowered by 2.7-fold, suggesting that MMP disruption is an inherent apoptotic mechanism. Polyphenols, which have a function in promoting oxidative stress in cancer cells, were shown to disrupt MMPs. As compared to the positive control H2O2, the DCFDA staining of cells showed almost 1.68 fold increased ROS levels in PC3 cells in the presence of CUR-B-CH-NPs. Bioenhancers P, N, and Z, on the other hand, has a negligible cytotoxic impact on PC-3 cells. Weak phytochemicals in combination have been shown in previous investigations to produce an outstanding synergistic cocktail (260). According to *in-vivo* results, CUR-B-CH-NPs displayed a markedly enhanced bioavailability compared to CUR-bioenhancement combo CZP/CUR solution, which is well expected. Relative to CZP and CUR solution, CUR-B-CH-NPs have significantly higher AUC by 4 and 6 times, as well as Cmax by 1.9 and 7.7 times, respectively. This may be due to the chitosan protecting CZP, which protects the drug from acidic degradation and also facilitates controlled release with the help of surfactant pluronic F68, according to the detailed statistical data. By enhancing intestinal membrane permeability and CZP cellular internalisation in CH-NPs1, Pluronic F68 improves CUR solubility.

Yallupu et al., developed curcumin-PLGA based nanoparticles for PCa (261). The internalisation of PLGA-CUR NPs was time-dependent. There was a significant amount of NPs in membrane vesicles during the first hour, and by the end of the second hour, NPs have completely entered the cells. NPs were found around the nucleus at the 18-hour mark. Based on the substantial internalisation and distribution pattern of NPs, the endocytosis/phagocytosis mechanism is likely clathrin-mediated. After being endocytosed, nanoformulations may be released from the endosome and end up in the nucleus or cytoplasm. Flow cytometry demonstrated the absorption of PLGA-CUR NPs at the 18-hour time point, with obvious cellular localisation. Variable PC cells have different levels of PLGA-CUR NP endocytosis. In the three studied PC cell lines, there may be variances in the method of internalisation of PLGA-CUR NPs because of the existence of different lipid membranes on their surfaces.Long-term accumulation and retention, in addition to internalisation, are critical for improving the therapeutic effectiveness of cancer medicines. Rapid breakdown or cellular export of many cancer medicines prevents therapeutic levels from reaching cells. PLGA-CUR NPs showed increased accumulation and retention compared to free CUR at each time point. All three cell lines examined retained a different amount of DNA, which was in keeping with the results from the TEM. The concentration of DU-145 cells peaked on day 2 and decreased dramatically on day 4. Day 1 to 2 saw an increase in C4-2 cells, which remained essentially constant at day 4, but day 3 to 4 saw a decrease. Free curcumin and PLGA-CUR NPs were substantially less stable in PC-3 than in PC-1, with a peak accumulation on day 2. Varied cancer cell types may respond to PLGA-CUR NPs differently because of their different cellular levels. Both C4-2 and DU-145 cancer cells produced a significant number of vacuoles under TEM. Lysosomal activity was also found to be abnormally high in this group of cells, which may have resulted from membrane instability that causes cell death/apoptosis signals. All of the vacuoles were shown to leak bioactive CUR from the nucleus’s periphery when NPs are internalised (262). By causing vacuoles in the cell body, the presence of PLGA-CUR NPs in PC cells led in cell membrane protrusion and disruption of the cytoskeleton (263). PC cells were not affected by the vacuolization induced by free CUR (264). Smaller and fewer cysts in PC-3 cells imply less absorption of nanoparticles than larger and larger vacuoles.

Cell death in PC cells was enhanced by PLGA-CUR nanoparticles, which promoted PARP cleavage and reduced the expression of anti-apoptotic proteins such Bcl-xL and Mcl-1 (265). Cleaved PARP plays a vital role in the activation of Caspase-3/7, which leads to apoptosis (265). When Mcl-1 and Bcl-xL are downregulated, platelet-derived growth factors (PDGF) and β-catenin transcription factors (TCF) are suppressed. On the other hand, the expression of AR and beta-catenin in cells was shown to be inhibited by the PLGA-CUR NPs. β-catenin is a multifunctional protein that plays a critical role in both ontogenesis and oncogenesis. Increased AR activation has been linked to β-catenin dysregulation (266) and the development of various malignancies, including PC. The upregulation of PKD1 by PLGA-CUR which has been shown to suppress the production of nuclear β-catenin and AR (267, 268).

In 2017, Azandeh et al. published a complementary research on PC-3 PC cells treated with Cur-PLGA NPs (269). When curcumin was applied to PC-3 cells, cell viability and proliferation decreased, with a higher loss in cell viability for Cur-PLGA NP-treated cells than for Cur-treated cells. PC-3 cell growth was dramatically decreased by curcumin-PLGA NPs, but PNT2 (healthy) cells were unaffected by treatment with curcumin. It was determined that Cur-PLGANPs have affected the chromatin structure of PC-3 cells, but PNT2 cells did not respond to treatment with the Cur-PLGA NPs. Annexin V/PI staining also showed that cells treated with Cur-PLGA NP having greater apoptosis and necrosis index. This therapy is promising for future investigations against PC since it induced cell death *via* both types I (apoptosis) and type II (autophagy/necrosis) of programmed cell death (269).

Anitha et al., formulated water soluble O-carboxymethyl chitosan based curcumin nanoparticles(O-CMC Nps) (270). Curcumin encapsulation and loading efficiency in O-CMC Nps were determined to be 87% and 48%, respectively. Results showed that the drug loading was greatly affected by the concentrations of O-CMC and curcumin. The higher the O-CMC concentration, the larger the particles were. Where the drug tends to precipitate, trapping efficiency decreased with greater drug concentrations. Curcumin and curcumin-O-CMC Nps in the dose range of 1–5 mg/ml decreased the viability of L929 but not curcumin alone when exposed to O-CMC Nps. Curcumin, O-CMC Nps, and curcumin-O-CMC Nps were not hazardous to normal cells, as shown by the fact that 80% of the cells were alive. MCF-7 exhibited no toxicity for O-CMC Nps whereas curcumin and curcumin-O CMC Nps showed significant toxicity. A similar toxicity was seen in PC-3 wherein cell viability was decreased to 30% at 5 mg/ml, confirming its anticancer properties. Curcumin-O-CMC Nps and curcumin has a similar impact on cancer cells, indicating that curcumin maintains its anticancer action even after being placed into a polymer matrix. Increases in curcumin-O-CMC Nps concentrations boost the absorption in both cell lines, according to the uptake profile.The negative charge of curcumin-O-CMC Nps may explain the lack of variation in particle absorption between normal and malignant cell lines. Cellular absorption is non-specific and concentration-dependent, according to this study’s findings. O-CMC Nps did not cause apoptosis in both cancerous and non-cancerous cells exposed to it. Compared to L929, MCF-7 has a higher proportion of apoptotic cells, which is obvious. The greater dose of curcumin-O-CMC Nps (5 mg/ml) resulted in a larger percentage of cells showing apoptosis than the lower quantity (1 mg/ml). Curcumin-O-CMC Nps may have been more hazardous to cancer cells at greater concentrations because of the increased absorption of curcumin-O-CMC Nps. In spite of the fact that normal and cancer cells have a similar absorption rate of particles, the greater apoptosis in cancer cells suggests the release of curcumin inside cancer cells and demonstrates the drug’s particular anticancer effect.” One explanation for this is curcumin’s ability to target signalling molecules found in high concentrations in cancer cells. Curcumin stops cell division in G0 phase without causing apoptosis in normal cells since these mechanisms are controlled.

Vodnik et al., developed Genistein (Gen) stabilized gold nanoparticles for targeting PCa (271). TEM was able to characterise the shape, size, and distribution of well-dispersed AuNPs when Gen was used as a reducing and capping agent in the same molecule. Capped Gen-coated AuNPs are round, and their size distribution is restricted. For Gen@AuNPs1 and Gen@AuNPs2, the average particle diameter (dav) was determined to be 10 nm and 23 nm, respectively. As a consequence of the greater Gen and Au^3+^ concentrations added, the second conjugate grew in diameter. In cell culture, treatment with Gen resulted in a dose-dependent reduction in cell numbers, as measured by mitochondrial respiration. It was found that the half-life values determined for PC3 cells and DU 145 cells were quite close (21.0 +/-0.6 and 22.3 +/-1.3), but LNCaP cells were more susceptible (13.9 +/-0.8). These two cell lines, DU 145 and PC3, are more aggressive than LNCaP, which explains their lower response. Considering that less than 50% of Gen was loaded onto each AuNP, it is clear that binding to AuNPs increased Gen’s cytotoxicity. Gene concentrations at 9 and 14 g/mL were measured in LNCaP cells using Gen@AuNPs1 (46 percent Gen loaded onto AuNPs) and Gen@AuNPs2 (48 percent loaded Gen), respectively, according to the proportion of loaded Gen. Even while Gen’s anticancer potential isn’t much boosted, the improved stability and distribution of Gen in this formulation may be critical for its continuing use *in vivo*. Both early and late apoptosis were not seen when Gen was incubated with free Gen or Gen@AuNPs1 formulations. Autophagy was likewise unaffected by both treatments, demonstrating that experimental therapies do not rely on programmed cell death type II as a cytotoxic mechanism (272). There was a considerable reduction in viability, however, since cell multiplication was considerably suppressed. By affecting the production of the human telomerase reverse transcriptase and different microRNAs, genistein has been shown to decrease PC cell growth (273, 274).

As a result, these anticancer nano-formulations are capable of increasing medication release and activation inside tumors, enhancing the therapeutic efficiency of the treatments. Tumor microenvironment features including hypoxia, acidity, the EPR effect, the presence of proteolytic enzymes, and the overexpression of certain cell membrane antigens or proteolytic factors may all cause disease-specific activation in solid tumors. As a result of the presence of these tumor-specific endogenous properties, it is possible to create formulations that are more active in the tumor microenvironment. When it comes to medication delivery strategies for cancer, polyglutamate (PGA) is one of the biodegradable polymers that have proved effective (275). However, PGA may be digested by cathepsin-B, which is released into the tumor microenvironment of most solid tumors and is lysosomal protease cathepsin-B. Results show that cathepsin B-induced digestion of nanoparticles into smaller particles might result in better dispersion of the sensitizer in dense tumor formations, as shown by this study (276). Because of the high interstitial fluid pressure and the thick network of collagen fibres, nanoparticle transport is impeded in dense tumor masses (277). Nanoparticles may be digested into smaller particles by the enhanced pericellular cathepsin B released by malignant tumors, according to the findings of this study. To reduce perivascular sequestration and trapping, this may help increase particle diffusion across the dense tumor mass during extravasation. lysosomes are predicted to be the site where cathepsin B digests nanoparticles completely and releases hematoporphyrin. The sonochemical effects of ultrasonic irradiation may stimulate the inclusion of free hematoporphyrin or amphiphilic hematoporphyrin complexes in the lysosomal membrane, the sensitization of the latter and the subsequent lysosome collapse (278). Previously, it has been demonstrated that lysosomal collapse may cause to apoptosis *via* lowering cytoplasmic pH. As a result, the treatment modality presented in this work is speculated to have a plausible mechanism of action, and the effect is supported by the nanoparticulate formulation’s responsiveness to cathepsin B. Although hydrogen peroxide is not a typical harmful ROS produced during sonodynamic activation utilising the nanoparticulate formulation, the ROS-induced DPBF breakdown and subsequent drop in absorbance were both shown to occur over the course of five minutes. Additionally, ROS generation was compared when hematoporphyrin was free and when no sensitising agent was present, namely just when ultrasonic irradiation was used. Nanoformulations show equivalent efficacy to free hematoporphyrin (p > 0.05) in terms of ROS generation, which is suggestive of an effective SDT-induced antitumor impact. As a result of this research, a nanoparticulate formulation has been created that specifically targets the acidic tumor interstium and cathepsin B. Cathepsin B is a proteolytic enzyme common in malignant tumor microenvironments, and cancer cells modulate its production and release extensively based on interstitial pH. This enzyme’s intracellular and secreted levels in LNCaP cells were examined as a critical first step in our attempt to use cathepsin B to enhance our therapeutic platform’s performance in SDT. The incubations were carried out under hypoxic circumstances, at 2 mmHg O_2_, since hypoxia is known to activate cathepsin B production in tumors (279). As compared to pH 7.4, levels of cathepsin B in LNCaP cells at pH 6.4 were 35 percent higher and 61 percent higher than those at pH 7.4. Finally, cellular absorption of 5-g/mL nanoparticles was measured for both pH conditions at final concentrations of 5 g/mL. For pH 6.4, HPNP absorption by LNCaP cells was enhanced by 75%, and this corresponds to an increase in cathepsin B. Although there isn’t conclusive proof of a link between cathepsin B levels and cellular absorption of PGATyr-based nanoparticles, it does show that the proteolytic enzyme’s levels influence cellular uptake. These findings may in part be owing to an enhanced protonation under acidic conditions of the glutamate residue side chains, which would contribute to an improvement in cell internalisation. HP and PGATyr co-polymer have been used to generate a nanoparticle formulation. To find out how well the nanoparticles performed when exposed to pH and cathepsin B, researchers examined the nanoparticles’ reactivity to each. According to the research findings, cathepsin B digestion reduced the size and overall negative charge of the formulation’s nanoparticles, perhaps allowing for better nanoparticle diffusion into impenetrable tumor tissues after extrusion. For LNCaP and PC3, the “silent” toxicity profiles were different, although the acidic pH increased the nanoparticle toxicity for both cell lines. Cellular absorption seemed to be inversely related to the production and secretion of cathepsin B. For PC cells treated with HPNP and treated *in vitro* with sonodynamic therapy, the stimulus (ultrasound) and formulation has little or no impact when not combined. Compared to the free sensitizer, the nanoparticulate formulation considerably increased HP’s sonodynamic activity in cell-based systems, principally due to better cellular absorption of the HP nanoparticles. SDT therapy in immunodeficient mice resulted in a 36% decrease in LNCaP tumor sizes after 24 hours of administration of a single dose of nanoparticles. There were no detrimental effects on the nanoparticle-treated animals, and their weight remained steady.

### 4.3 Micellaenous

Since quercetin has a low bioavailability in castration-resistant PC, Zhao et al. used nano micelles to encapsulate it for *in vitro* and *in vivo* research (280). Quercetin’s water solubility was increased 450-fold by encapsulating at 1 mg/mL. According to the results of the *in vitro* investigations, micellar quercetin formulation has a half maximal inhibitory concentration of 20 M, whereas free quercetin has noticed a concentration of 200 M. As a result, the nano based formulation effectively triggered apoptosis and suppressed cell growth in human androgen prostate cancer cell lines. In addition, quercetin-loaded micelles *in vivo* showed greater anticancer activity, and the proliferation rate dropped by 52% compared to the control group in the PC-3 xenograft mouse model, possibly owing to higher permeability and retention of micellar quercetin at the tumor site.

Cancer relapse, chemoresistance, and recurrence are all made more likely by cellular senescence, a persistent problem in cancer treatment. Drug-induced senescence is a common side effect of long-term chemotherapy treatment. It is well-known that Docetaxel, a prostate cancer medication authorised by the FDA, may cause cellular senescence, reducing the overall survival time of patients. Anti-aging strategies for cells and drugs are still unmet therapeutic needs. With the goal of developing an innovative therapy that targets and destroys senescent cells, researchers created a nanoformulation of tannic acid–docetaxel self-assemblies in an attempt to achieve this goal (DSAs) (281). Particle size, spectroscopic, thermal, and biocompatibility investigations verified the creation of DSAs. Docetaxel was shown to be more effective in this formulation when compared to docetaxel alone in a variety of biological functional tests. Senescence-associated TGFR1/FOXO1/p21 signalling was altered by DSAs exposure, according to microarray and immunoblot research findings. After DSAs exposure, a decrease in -galactosidase staining indicated a reversal of drug-induced senescence. In addition, DSAs triggered apoptosis by circumventing senescence, resulting in a dramatic increase in cell death. In addition, imaging studies in mice with PC-3 xenograft tumors showed that DSAs target tumors both *in vivo* and ex vivo. Using the PC-3 xenograft mouse model, the antisenescence and anticancer effect of DSAs was shown by suppressing TGFR1 proteins and regressing tumor development *via* apoptosis. These enhanced properties of DSAs were all attributed to the use a natural substance as the matrix/binder for docetaxel in the formulation. Docetaxel effectiveness was enhanced by DSAs’ greater tumor targeting and increased cell internalisation. Prostate cancer treatment may benefit greatly from these discoveries.

Radhakrishnan et al. developed epigallocatechin-3-gallate (EGCG) loaded solid lipid nanoparticles (SLN) by double emulsification method to enhance the stability and anticancer efficacy of loaded phytoconstituents (282). The *in vitro* cytotoxicity was performed by MTT assay using breast cancer, and prostate cancer cell line (MDA MB-231, and DU-145 respectively), where EGCG-SLN showed increase in cytotoxicity against (8.1 fold) MDA MB-231 and (3.8-fold) DU-145 cell lines. In a colloidal stability study, stability with high resistance to electrolyte synthesis was observed in both serum and P135. They concluded that EGCG-SLN acts as a promising nanocarrier for delivering epigallocatechin-3-gallate as a powerful anticancer agent.

Khan et al. suggested a different approach involving the nanoencapsulation of epigallocatechin-3-gallate (EGCG) by oral administration to treat prostate cancer (283). Chit-nano EGCG performed kinetic experiments, in which the release of EGCG has minimal effects of mimicking gut juice simultaneously while EGCG was able to be released rapidly. The effectiveness of the antitumor Chit-nano EGCG was tested by implanting 22Rv1 xenografts under the skin in naked Athymic mice. By comparing the tumor tissue of mice treated with chit-nano EGCG to the EGCG control groups, significant effects were observed as cracking of ADP-ribose polymerase induction, increased Bax protein exposure and a corresponding decrease in Bcl-2 expression. Activation of caspases, reduction of Ki-67 and proliferation of cell nuclear antigen. Through this study, they came to the conclusion that EGCG acts as a preventive and curative agent for prostate cancer.

Blanco et al., prepared β-Lapachone (β-lap) loaded PEG-PLA polymeric micelles to treat quinone oxidoreductase 1 (NQO1) overexpressing tumors (284). β-Lapachone is a chemotherapeutic agent, biologically activated by NADP(H): quinone oxidoreductase 1 coenzyme which is overexpressed in most of the tumor cells. To increase the loading efficiency of micelles dialysis, solvent evaporation and sonication method are used in which film sonication method yielded maximum loading density (4.7 ± 1.0% to 6.5 ± 1.0) with optimum size of 29.6 ± 1.5 nm. They conducted a drug release kinetic study of polymeric micelles, which shows 50% drug release within 18 h of time period. The *in vitro* cytotoxicity study was conducted on lung, prostate, and breast cancer cell lines (NQO1-overexpressing (NQO1 +) and NQO1-null H596, DU-145, and MDA-MB-231 cell lines). The results of cytotoxicity showed increase in toxicity on NQO1+ cells over NQO1- cells anticipating β-Lapachone micelles as a promising nanocarrier against NQO1-overexpressing tumor cells.

Mukerjee et al., prepared PLGA nanosphere by solvent evaporation technique to check the potent anticancer effect of curcumin (262). The study’s primary objective was to mask the demerits of curcumin associated with low oral bioavailability and poor aqueous solubility. The particle size of the nanosphere was within the range of 35 to 100 nm, with the mean size of 45 nm. PLGA nanosphere showed encapsulation efficiency of 90.88 ± 0.14%. The cellular viability of curcumin PLGA nanosphere was evaluated on prostate cancer cell line where it showed more pronounced effect when compared with free curcumin. These results of the PLGA nanosphere were more promising, and as adjuvant therapy, it can be used to treat prostate cancer.

## 5 Nanotoxicity of Nanomaterials

Currently, nanomaterials are pervasive in everyday life (285). This toxicity and the destiny of nanomaterials depends on their physical and chemical characteristics, which are determined by their usage in everyday life. The physiochemical features of nanomaterials, such as charge, surface area, shape, size, and aggregation, are unique to this kind of material. Nanomaterials are more reactive than bulk materials because of their tiny size, and their ability to enter cells and cause toxicity is also higher. Smaller nanomaterials are more harmful than larger ones because they are easier to get into the body’s organs. Reactive oxygen species (ROS) are formed when nanomaterials are retained in organs (286).

Toxicities associated with nanomaterials may be classified according to their described morphologies, which include rods, cubes, ellipsoids, spheres, and cylinders (287). Surface chemistry characteristics as roughness, charge, and hydrophobicity may have a substantial impact on the toxicological consequences of nanomaterials. It is possible that nanoparticles’ surface features impact the blood-brain barrier and the immune system, as well as the phagocytosis, colloidal behavior, and cellular absorption of nanomaterials. Compared to negatively charged nanoparticles, positively charged nanomaterials have a far greater absorption rate Once nanomaterials permeate membranes and bind tightly to DNA because it is negatively charged, the G0/G1 stages of cell life cycles are prolonged. The positively charged nanomaterials have a stronger affinity for proteins and may modify protein structure, which may lead to the suppression of enzyme activities and the subsequent disruption of biological processes (288).

Materials having cationic surface charges are more likely than neutral or anionic nanoparticles to interact with genetic materials and biological membranes, resulting in greater toxicity (289). Agglomeration and aggregation of nanomaterials, as well as surface charge and size, may change the blood-brain barrier’s integrity (290). Nanomaterials may be made of inorganic or organic components, and a large number of reagents are needed to make them. As a consequence, unanticipated toxicity and side effects may occur owing to the presence of contaminants or other undesired components. The body’s nanomaterial composition may alter due to internal pH and oxidation reduction reaction variations (291).

For nanotoxicity, the solubility of nanomaterials is a key issue. The dissolution of nanomaterials is influenced significantly by temperature and pH fluctuations. Unlike insoluble nanoparticles, soluble nanomaterials may be very hazardous. Even though nanoparticles have diverse physiochemical characteristics, agglomeration may produce considerable toxicity and increasing exposure levels to nanomaterials might induce chronic illnesses including cancer and fibrosis. Nanotoxicology relies heavily on nanoparticles’ morphology. According to Firme III and Bandaru (292),, long-term inhalation of nanofibers or nanomaterials may lead to lung inflammation and cancer. It’s been shown *via* several research that carbon nanotubes are much more hazardous to health than either silica dust or ultrafine carbon black (293).

When nanomaterials are dispersed and agglomerated, their toxicity is directly affected by the circumstances in which they are dispersed and agglomerated. Additionally, the hazardous effects of nano-materials are influenced by the media in which they are dispersed. Using the same nanomaterials in a variety of different environments results in various harmful effects (294). Some of the dispersion agents may increase the physical and chemical characteristics of nanomaterials in the medium, while others can have detrimental impacts on the environment, resulting in hazardous consequences.

In terms of toxicity, nanoparticles are mostly determined by their surface characteristics. The physicochemical features of nanomaterials, such as their chemical reactivity and their optical, magnetic, and electrical properties, may be modified by surface coating (295).

For nanoparticles, researchers have developed a risk assessment framework that incorporates alternative testing methods for individual nanomaterials. However, animal model testing is significantly reliant on most testing methods for assessing the toxicity of nanomaterials (296). Due to the fact that nanomaterials interact with the human body in a unique way, further study is needed to establish a long-term and effective solution.

## 6 Conclusion and Future Directions

Toxicities, poor bioavailability, limited selectivity, and multidrug resistance have all been addressed using nanotechnology in cancer therapy. Chemotherapy’s non-specificity has long destroyed normal growing tissues in patients, causing immunodeficiency and long-term adverse effects. Nanotechnology has offered powerful treatment techniques due to its selectivity to target malignant cells. Patients treated with nanoparticles had improved therapy response and long-term survival. Nanomedicine in cancer treatment may therefore easily solve the obstacles hampering traditional therapy regimens. Developing patient-specific medication delivery systems would assist personalise and manage therapy depending on the patient’s clinical profile. Nanomedicine promises to be the next worldwide transformation in cancer therapies, allowing for early tumor detection and patient management.

To prevent tumor formation or reduce cancer incidence, chemopreventive medicines are much sought after. Because the current therapeutic options include chemotherapy, radiation, and surgery, which all have considerable adverse effects, alternative or adjuvant treatments are urgently needed. Phytochemicals are harmless and plentiful in foods. So, alternative medicine attempts to use these non-essential nutrients to prevent and cure cancer. Many studies support the use of biomolecules in cancer therapy, however most are *in vitro*. Despite few *in vivo* and clinical trials, phytochemicals offer considerable potential in cancer therapy. Efficacy of these compounds in clinical studies must be approached with caution as numerous variables influence their biological effects. This is particularly true when low nontoxic dosages are necessary for lengthy durations to achieve significant chemotherapeutic results with minimum adverse effects. Dosage and delivery are now important issues. To maintain a consistent physiological serum dosage availability, the agent must be concentrated and stable in the target tissue. Combination technology may solve this issue. Nanotechnology is rapidly becoming the next level of scientific technology. *In vitro* research have showed that encapsulating dietary supplements in nanoparticles increases their delivery, stability, and availability. Maybe studies should look at employing combo therapies. Given the findings in this analysis, it will be fascinating to gather further pre-clinical evidence on these compounds’ anticancer properties. Although little is known regarding natural chemical bioavailability *in vivo*. However, further high-quality research are required to conclusively confirm plant extracts’ therapeutic usefulness, solely and as synergisticaly.

## Author Contributions

Conceptualization, HC and TE; data curation, HC, SB, and RaG; writing —original draft preparation, HC, RaG, RuG, RT, TU, MM, MS, and MH; validation, KK, GG, IS, JK, JJ, MA-D, FA, TE, and BK; formal analysis, KK, GG, IS, JK, JJ, MA-D, FA, TE, and BK; investigation, HC, RaG, RuG, RT, TU, MM, MS, and MH; resources, HC and TE; writing—reviewing and editing, KK, GG, IS, JK, JJ, TE, and BK;; visualization, KK, GG, IS, JK, JJ, MA-D, FA, TE, and BK; supervision, SB, JJ, TE, and BK; project administration, SB, JJ, TE, and BK; funding acquisition, TE and BK;. All authors have read and agreed to the published version of the manuscript.

## Funding

This research was supported by Basic Science Research Program through the National Research Foundation of Korea (NRF) funded by the Ministry of Education (NRF-2020R1I1A2066868), the National Research Foundation of Korea (NRF) grant funded by the Korea government (MSIT) (No. 2020R1A5A2019413), a grant of the Korea Health Technology R&D Project through the Korea Health Industry Development Institute (KHIDI), funded by the Ministry of Health & Welfare, Republic of Korea (grant number: HF20C0116), and a grant of the Korea Health Technology R&D Project through the Korea Health Industry Development Institute (KHIDI), funded by the Ministry of Health & Welfare, Republic of Korea (grant number: HF20C0038).

## Conflict of Interest

The authors declare that the research was conducted in the absence of any commercial or financial relationships that could be construed as a potential conflict of interest.

## Publisher’s Note

All claims expressed in this article are solely those of the authors and do not necessarily represent those of their affiliated organizations, or those of the publisher, the editors and the reviewers. Any product that may be evaluated in this article, or claim that may be made by its manufacturer, is not guaranteed or endorsed by the publisher.
